# Point-of-care testing for emergency assessment of coagulation in patients treated with direct oral anticoagulants

**DOI:** 10.1186/s13054-017-1619-z

**Published:** 2017-02-15

**Authors:** Matthias Ebner, Ingvild Birschmann, Andreas Peter, Charlotte Spencer, Florian Härtig, Joachim Kuhn, Gunnar Blumenstock, Christine S. Zuern, Ulf Ziemann, Sven Poli

**Affiliations:** 1 Department of Neurology & Stroke, and Hertie Institute for Clinical Brain Research, University of Tübingen, Tübingen, Germany; 2 Department of Internal Medicine and Cardiology, Charité University Medicine Berlin - Campus Virchow Klinikum, Berlin, Germany; 3 Institute for Laboratory and Transfusion Medicine, Heart and Diabetes Center, Bad Oeynhausen, Ruhr University, Bochum, Germany; 4 Department of Internal Medicine, Division of Endocrinology, Diabetology, Angiology, Nephrology and Clinical Chemistry, University of Tübingen, Tübingen, Germany; 5 Institute for Diabetes Research and Metabolic Diseases of the Helmholtz Center Munich at the University of Tübingen, Tübingen, Germany; 6 German Center for Diabetes Research (DZD), Tübingen, Germany; 7 Department of Clinical Epidemiology and Applied Biometry, University of Tübingen, Tübingen, Germany; 8 Department of Cardiology and Cardiovascular Medicine, University of Tübingen, Tübingen, Germany

**Keywords:** Point-of-care testing, POCT, Direct oral anticoagulants, DOAC, Non-vitamin K antagonist oral anticoagulants, NOAC, Emergency surgery, Anticoagulation reversal, Thrombolysis, Stroke

## Abstract

**Background:**

Point-of-care testing (POCT) of coagulation has been proven to be of great value in accelerating emergency treatment. Specific POCT for direct oral anticoagulants (DOAC) is not available, but the effects of DOAC on established POCT have been described. We aimed to determine the diagnostic accuracy of Hemochron® Signature coagulation POCT to qualitatively rule out relevant concentrations of apixaban, rivaroxaban, and dabigatran in real-life patients.

**Methods:**

We enrolled 68 patients receiving apixaban, rivaroxaban, or dabigatran and obtained blood samples at six pre-specified time points. Coagulation testing was performed using prothrombin time/international normalized ratio (PT/INR), activated partial thromboplastin time (aPTT), and activated clotting time (ACT+ and ACT-low range) POCT cards. For comparison, laboratory-based assays of diluted thrombin time (Hemoclot) and anti-Xa activity were conducted. DOAC concentrations were determined by liquid chromatography-tandem mass spectrometry.

**Results:**

Four hundred and three samples were collected. POCT results of PT/INR and ACT+ correlated with both rivaroxaban and dabigatran concentrations. Insufficient correlation was found for apixaban. Rivaroxaban concentrations at <30 and <100 ng/mL were detected with >95% specificity at PT/INR POCT ≤1.0 and ≤1.1 and ACT+ POCT ≤120 and ≤130 s. Dabigatran concentrations at <30 and <50 ng/mL were detected with >95% specificity at PT/INR POCT ≤1.1 and ≤1.2 and ACT+ POCT ≤100 s.

**Conclusions:**

Hemochron® Signature POCT can be a fast and reliable alternative for guiding emergency treatment during rivaroxaban and dabigatran therapy. It allows the rapid identification of a relevant fraction of patients that can be treated immediately without the need to await the results of much slower laboratory-based coagulation tests.

**Trial registration:**

Unique identifier, NCT02371070. Retrospectively registered on 18 February 2015.

**Electronic supplementary material:**

The online version of this article (doi:10.1186/s13054-017-1619-z) contains supplementary material, which is available to authorized users.

## Background

Direct oral anticoagulants (DOAC) are being increasingly prescribed. Consequentially, more DOAC-treated patients are admitted to emergency units across all clinical specialties. Importantly, emergency coagulation testing in DOAC-treated patients has not been solved, which complicates emergency treatment decisions such as emergency surgery, anticoagulation reversal, and thrombolysis in ischaemic stroke.

In patients receiving vitamin K antagonists (VKA), point-of-care testing (POCT) of coagulation has proven its great value in accelerating emergency treatment [1]. Although DOAC-specific POCT is not currently available, results obtained by our group suggest that relevant plasma concentrations of rivaroxaban can be qualitatively ruled out with CoaguChek® (Roche, Basel, Switzerland), a prothrombin time/international normalized ratio (PT/INR) POCT designed for VKA monitoring [2]. However, CoaguChek® lacked sensitivity to apixaban and dabigatran.

Hemochron® Signature (ITC, Edison, NJ, USA) is another coagulation POCT that uses a different PT/INR assay to CoaguChek® and has additional measuring capability for activated partial thromboplastin time (aPTT) and activated clotting time (ACT). Previous reports indicate that Hemochron® Signature is responsive to rivaroxaban and dabigatran [3–7]. However, these reports aimed at quantitative measurements rather than qualitatively ruling out low but relevant DOAC concentrations, and used either artificially DOAC-spiked plasma samples [3, 4] or they only comprised a few samples with low DOAC concentrations [5–7]. Furthermore, the utility of Hemochron® Signature to measure the effects of apixaban on coagulation effects has never been reported. Thus, the diagnostic value of Hemochron® Signature in guiding emergency treatment decisions in DOAC-treated patients has not been clarified.

## Methods

### Study aim and design

We aimed to determine the diagnostic accuracy of Hemochron® Signature to qualitatively rule out relevant concentrations of apixaban, rivaroxaban, and dabigatran in real-life patients.

The study was a single-centre, prospective observational trial with blinded end-point assessment. The Clinical Trial Registration Information unique identifier is NCT02371070.

### Setting and eligibility criteria

The study was conducted at the Department of Neurology & Stroke and the Department of Cardiology and Cardiovascular Medicine of Tübingen University Hospital, Tübingen, Germany, a tertiary care facility. Patients receiving first doses of apixaban, rivaroxaban, or dabigatran were enrolled. Exclusion criteria were known coagulopathy, abnormal coagulation at baseline (PT >13 s/Quick <70%/INR >1.2 or aPTT >37 s), intake of VKA or DOAC within 14 days, low-molecular-weight heparins within 24 h or unfractionated heparin within 12 h prior to DOAC intake. Use of antiplatelet agents was permitted.

Predominantly low dabigatran concentrations in these samples required inclusion of additional samples from patients on maintenance therapy with dabigatran. The same exclusion criteria as above applied, except that abnormal coagulation at baseline (due to dabigatran intake) was allowed.

### Sample collection

Six blood samples were collected from each subject via an indwelling venous catheter or venipuncture: before DOAC intake, 30 min and 1, 2, and 8 h after intake, and at trough (12 h for apixaban and dabigatran, 24 h for rivaroxaban).

### POCT and laboratory-based coagulation testing

Bedside POCT of PT/INR, aPTT and ACT was conducted from whole blood. ACT was measured using ACT plus (ACT+) and ACT-low range (ACT-LR) test cards that contain different reagents and cover different measurement ranges. Further samples were collected in 3.2% sodium-citrate tubes (Sarstedt, Nümbrecht, Germany), and instantly centrifuged to acquire plasma. Laboratory-based anti-Xa activity was measured using Chromogenix COAMATIC Heparin Test on an ACL TOP 700 (Instrumentation Laboratory, Kirchheim, Germany). TECHNOVIEW calibrators (Technoclone, Vienna, Austria) were used to determine apixaban and rivaroxaban concentrations with limits of quantification of 10 and 18 ng/mL. Remaining plasma aliquots were stored at –80 °C until testing of diluted thrombin time (dTT; Hemoclot assay, Hyphen BioMed, Neuville-sur-Oise, France). As the gold standard, DOAC concentrations were determined by ultra-performance liquid chromatography-tandem mass spectrometry (UPLC-MS) [8]. All coagulation testing was performed according to manufacturers’ instructions by thoroughly trained investigators and technicians.

### Blinding

All laboratory-based tests, including UPLC-MS, were conducted and interpreted by technicians blinded to POCT results.

### Definition of relevant DOAC concentrations

Our analyses were based on two likely emergency scenarios (Table 1). First, we investigated if POCT can be used to detect DOAC concentrations proposed as safe for surgical procedures [9–12]. As two of the proposed safe-for-treatment thresholds for dabigatran are practically identical (<48 ng/mL and <50 ng/mL), we chose to evaluate the <50 ng/mL threshold only. For apixaban a concentration threshold that permits surgery is currently unknown [13]. Second, we evaluated concentrations that may allow thrombolysis in ischaemic stroke according to an expert recommendation [14].

**Table 1:** Investigated concentration thresholds

Investigated concentrations	Rivaroxaban (ng/mL)	Apixaban (ng/mL)	Dabigatran (ng/mL)
DOAC concentrations proposed as safe for surgery [10–12]	30	NA	30, 48, 50
DOAC concentrations that may permit thrombolysis with rtPA [14]	100	10	50

*DOAC* direct oral anticoagulants, *NA* not available, *rtPA* recombinant tissue plasminogen activator

### Statistics

For test performance analyses, results of samples (all baseline samples, all samples per DOAC) were pooled in order to obtain a clinically relevant concentration spectrum. SPSS v23 (IBM, Armonk, NY, USA) was used for statistics. Confidence intervals for proportions, i.e. diagnostic sensitivity and specificity, were calculated according to the efficient-score method using the free online VassarStats Clinical Calculator 1 [15]. Pearson’s correlation coefficient was used to estimate the strength of correlations between coagulation assays and DOAC concentrations. Correlation strength was graded as proposed by Evans: <0.20 very weak, 0.20–0.39 weak, 0.40–0.59 moderate, 0.60–0.79 strong, and >0.80 very strong [16]. Fisher’s exact test was calculated to determine differences in the proportions of baseline POCT results between the study groups and to examine the association between coagulation test results and DOAC concentrations dichotomized to concentrations below and above the chosen safe-for-treatment thresholds (Table 1). Diagnostic accuracy of coagulation tests at different cut-off points was expressed in terms of sensitivity, specificity, positive predictive value (PPV), negative predictive value (NPV), and likelihood ratio. Sensitivity was defined as the percentage of samples with DOAC concentrations below the chosen safe-for-treatment threshold that were correctly identified as eligible for treatment. Correspondingly, specificity was defined as the percentage of samples with DOAC concentrations above the corresponding threshold that were correctly identified as not eligible. Specificity >95% was defined as sufficient for clinical application. PPV was defined as the percentage of samples with DOAC concentrations below the chosen safe-for-treatment threshold of all samples identified as eligible for treatment. NPV was defined as the percentage of samples with DOAC concentrations above the chosen safe-for-treatment threshold of all samples identified as not eligible for treatment. All DOAC concentrations are reported as median and interquartile range (IQR). Sensitivity and specificity are given with two-sided 95% confidence intervals.

## Results

We enrolled 60 patients (*n* = 20 per DOAC) receiving first doses of DOAC and eight patients on dabigatran maintenance therapy between February 2014 and November 2015. Patient characteristics and baseline laboratory values are provided as part of Additional file 1: Tables S1 and S2).

### Samples and DOAC concentrations

Of 408 planned samples, 403 were collected (apixaban *n* = 117, rivaroxaban *n* = 118, dabigatran *n* = 168). Five samples could not be analysed due to POCT malfunction.

DOAC were not detected by UPLC-MS in any baseline sample acquired before first DOAC intake (*n* = 60). Samples collected after DOAC intake contained a median concentration of 57.2 ng/mL apixaban (IQR 35.3–101.2, *n* = 97), 99.3 ng/mL rivaroxaban (IQR 25.7–184.3, *n* = 98), and 29.0 ng/mL dabigatran (IQR 10.7–72.3, *n* = 148). Median trough dabigatran concentrations were significantly lower after the first dose than during maintenance therapy (16.3 versus 81.8 ng/mL, *p* < 0.001).

### Correlation between POCT results and DOAC concentrations

**Fig. 1:**
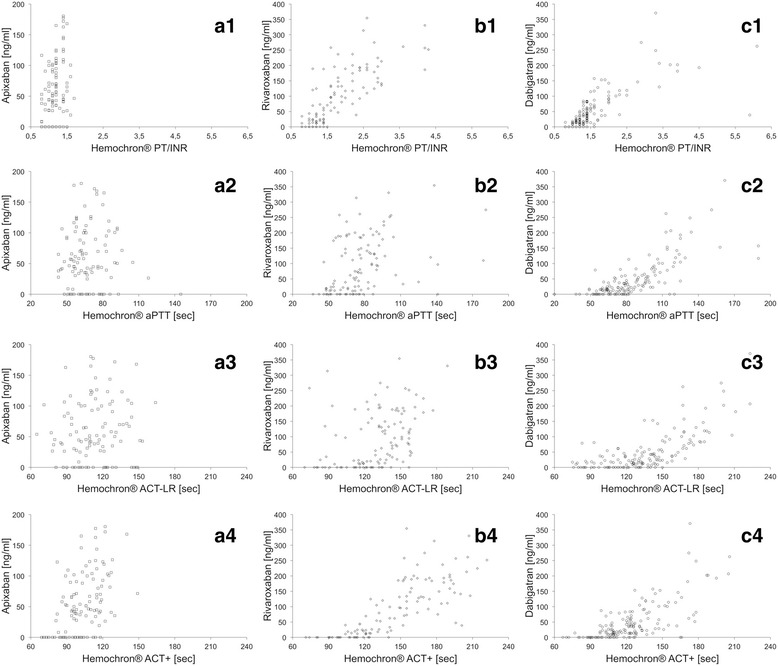
Hemochron® Signature POCT results (lines 1 to 4) plotted against concentrations of **a** apixaban, **b** rivaroxaban, and **c** dabigatran. Line 1: prothrombin time/international normalized ratio (*PT/INR*) POCT; line 2: activated partial thromboplastin time (*aPTT*) POCT; line 3: activated clotting time-low range (*ACT-LR*) POCT; line 4: ACT+ POCT. *n* = 118 samples for rivaroxaban, *n* = 117 samples for apixaban, and *n* = 168 samples for dabigatran

POCT of baseline samples before first DOAC intake showed no difference between DOAC groups. Correlations between POCT results and apixaban concentrations were weak for PT/INR (*r* = 0.37, *p* < 0.001) and moderate for ACT+ (*r* = 0.48, *p* < 0.001). No correlation was found for aPTT (*p* = 0.857) or ACT-LR (*p* = 0.174). For rivaroxaban, correlation was strong for PT/INR and ACT+ (*r* = 0.79 and 0.78, both *p* < 0.001) and weak for aPTT and ACT-LR (*r* = 0.399 and 0.383, both *p* <0.001). Dabigatran concentrations correlated strongly with all test cards (PT/INR, *r* = 0.75; aPTT, *r* = 0.75; ACT-LR, *r* = 0.69; ACT+, *r* = 0.67; all *p* < 0.001). Figure 1 shows the relations of the four POCT test cards to DOAC concentrations.

Due to insufficient correlation strength for apixaban, we limited all further analyses to rivaroxaban and dabigatran. To maximize clinical applicability, we focused on the two test cards that provided strong correlation to both rivaroxaban and dabigatran, i.e. PT/INR and ACT+.

### Diagnostic accuracy of POCT

**Fig. 2:**
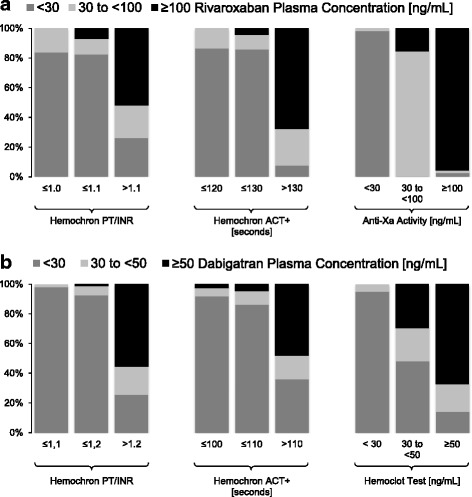
**a** Distribution of rivaroxaban concentrations found at different Hemochron® Signature POCT results of prothrombin time/international normalized ratio (*PT/INR*) and activated clotting time plus (*ACT+*) test cards and at different anti-Xa activities (*n* = 118 samples). **b** Distribution of dabigatran concentrations found at different Hemochron® Signature POCT results of PT/INR and ACT+ test cards, and at different Hemoclot assay results (*n* = 168 samples)

Rivaroxaban concentration was <30 ng/mL in 46/118 and <100 ng/mL in 79/118 samples. Dabigatran concentration was <30 ng/mL in 95/168 and <50 ng/mL in 117/168 samples. PT/INR and ACT+ POCT results for samples below and above each of the investigated safe-for-treatment thresholds differed significantly (all *p* < 0.001; Fig. 2). For reasons of clarity and comparison, test accuracy calculations are presented in Tables 2 and 3; additional calculations for aPTT and ACT-LR POCT cards are presented as part of Additional file 1: Table S3).

**Table 2:** Diagnostic accuracy of Hemochron® Signature POCT for rivaroxaban

Coagulation test result	Threshold (ng/mL)	Specificity (%)	Sensitivity (%)	LR	PPV (%)	NPV (%)
POCT PT ≤1.0	<30	97 (89–100)	33 (20–48)	11.6	88	69
POCT PT ≤1.1	<100	96 (85–99)	38 (27–50)	9.0	93	52
POCT ACT+ ≤120 s	<30	96 (87–99)	67 (52–80)	15.7	91	82
POCT ACT+ ≤130 s	<100	96 (85–99)	68 (55–78)	16.2	96	68

*n* = 118 samples

Sensitivity and specificity are provided with 95% confidence intervals

*ACT+* activated clotting time plus, *LR* likelihood ratio, *NPV* negative predictive value, *POCT* point of care test, *PPV* positive predictive value, *PT* prothrombin time

**Table 3:** Diagnostic accuracy of Hemochron® Signature POCT for dabigatran

Coagulation test result	Threshold (ng/mL)	Specificity (%)	Sensitivity (%)	LR	PPV (%)	NPV (%)
POCT PT ≤1.1	<30	99 (92–100)	47 (37–58)	34.6	98	59
POCT PT ≤1.2	<50	98 (88–100)	66 (56–74)	33.5	99	56
POCT ACT+ ≤100 s	<30	96 (87–99)	36 (26–47)	8.7	92	54
POCT ACT+ ≤100 s	<50	98 (88–100)	31 (23–40)	15.7	97	61

*n* = 168 samples

Sensitivity and specificity are provided with 95% confidence intervals

*ACT+* activated clotting time plus, *LR* likelihood ratio, *NPV* negative predictive value, *POCT* point of care test, *PPV* positive predictive value, *PT* prothrombin time

Rivaroxaban concentrations at <30 and <100 ng/mL were detected with >95% specificity at PT/INR POCT ≤1.0 and ≤1.1 and ACT+ POCT ≤120 and ≤130 s. Dabigatran concentrations at <30 and <50 ng/mL were detected with >95% specificity at PT/INR POCT ≤1.1 and ≤1.2 and ACT+ POCT ≤100 s (for both thresholds). Likelihood ratio (LR) was highest for rivaroxaban and ACT+ POCT (<30 ng/mL, LR 15.7; <100 ng/mL, LR 16.2), and dabigatran and PT POCT (<30 ng/mL, LR 34.6; <50 ng/mL, LR 33.5).

### Performance of laboratory-based DOAC-specific coagulation assays

The calibrated anti-Xa assay (Chromogenix COAMATIC Heparin Test) showed the highest correlation to rivaroxaban levels of all evaluated coagulation tests (*r* = 0.94, *p* < 0.001). Test results predicted rivaroxaban concentrations <30 and <100 ng/mL with a respective specificity of 99% and 92%, and a sensitivity of 98% and 97%.

Correlation between dTT (Hemoclot) and dabigatran concentrations was also very strong (*r* = 0.86, *p* < 0.001). Test results predicted dabigatran concentrations <30 and <50 ng/mL with a respective specificity of 95% and 84%, and a sensitivity of 77% and 82%.

## Discussion

### Summary

Results of Hemochron® Signature coagulation POCT correlate with rivaroxaban and dabigatran, but not with apixaban concentrations. PT/INR and ACT+ test cards are of particular interest, as they are influenced by both rivaroxaban and dabigatran. POCT-specific cut-offs can be used to exclude relevant rivaroxaban and dabigatran concentrations (Table 1) at the bedside with high specificity.

### Concentration thresholds

Data on what constitutes a relevant DOAC concentration, i.e. a concentration that leads to clinically significant coagulation impairment, are scarce. Current guidelines are predominantly time-based. Urgent surgical procedures may be performed at trough [9, 17], i.e. 12 or 24 h after last intake of apixaban/dabigatran [18, 19] or rivaroxaban [20], respectively. Thrombolysis for ischaemic stroke can be performed >48 h after the last intake of any DOAC [21]. However, significant DOAC concentrations have been observed in patients even beyond 48 h after the last intake [22]. Hence, the usefulness of time-based guidelines is limited. Furthermore, obtaining information on last DOAC intake might be impossible in patients with stroke and aphasia or dementia.

Specific concentration thresholds that increase bleeding risk can currently only be estimated based on retrospective analyses [10], pharmacodynamic considerations [11], and expert opinions [12, 14]. For surgical procedures, concentrations of rivaroxaban <30 ng/mL [10], and dabigatran <30 [10], <48 [11], and <50 ng/mL [12] have been proposed as safe. For apixaban, such concentration thresholds are currently unknown [13]. For acute ischaemic stroke, DOAC concentrations that may permit thrombolysis have been suggested by an expert group (see Table 1) [14]. It should be noted that these latter concentrations represent an expert opinion rather than quantitatively determined values.

Following these recommendations, we based our analysis on the concentrations proposed as safe for surgery [9–12] and the concentrations that may permit thrombolysis in ischaemic stroke [14].

### Coagulation testing for rivaroxaban and dabigatran

Quantification of DOAC concentrations using DOAC-specific assays is the recommended clinical standard for coagulation testing during DOAC treatment, i.e. anti-Xa activity for rivaroxaban and dTT or ecarin-based assays for dabigatran [17, 23]. Our study confirms that these assays provide high diagnostic accuracy for all tested DOAC. However, we found that Hemoclot had limited specificity and sensitivity for dabigatran concentrations <50 ng/mL, a threshold considered safe for surgery [11, 12] and thrombolysis [14]. At such low dabigatran concentrations, the reduced accuracy of Hemoclot has also been reported by others [24].

In many institutions, availability of DOAC-specific coagulation assays is limited [25]. For this reason, several authors have argued that unspecific global coagulation assays might suffice to rule out relevant DOAC concentrations [14, 26, 27].

Prompt availability and instantaneous results makes coagulation POCT an attractive option in emergency situations. We have previously shown that relevant concentrations of rivaroxaban can be qualitatively ruled out with CoaguChek®, a POCT designed for VKA monitoring [2]. The results of our current trial suggest Hemochron® Signature as a more versatile alternative: while CoaguChek® was only responsive to rivaroxaban, results of Hemochron® Signature PT/INR and ACT+ test cards were altered by both rivaroxaban and dabigatran.

Interestingly, the two factor Xa inhibitors, rivaroxaban and apixaban, differed in their effects on Hemochron® Signature POCT results. While POCT results at different apixaban concentrations were almost randomly distributed (Fig. 1), a strong correlation was found for rivaroxaban. Similar findings have been previously reported by our group and others for POCT and laboratory-based assays [2, 28]. To our knowledge, no convincing explanation for this difference has yet been provided.

Irregularities of Hemochron® Signature POCT results in the presence of dabigatran were noted early [4]. Recently, three trials evaluated this POCT (or the equivalent GEM® PCL Plus) for coagulation monitoring in patients treated with rivaroxaban [6], dabigatran [5], or both [7]. These studies reported that, in the case of low DOAC concentrations, coagulation test results frequently fell into a “normal range” previously established in untreated volunteers and concluded that the device cannot sufficiently distinguish between patients at trough concentrations and untreated patients [5–7]. We contest the usefulness of a “normal range” found in untreated volunteers for the monitoring of DOAC. In agreement with the previous reports, we observed that the alterations in coagulation results in the presence of DOAC often did not exceed values found in untreated patients. Nevertheless, our analyses show that POCT results below DOAC-specific cut-offs are highly predictive when used to qualitatively rule out relevant concentrations of rivaroxaban and dabigatran.

### Translation into the real world

**Fig. 3:**
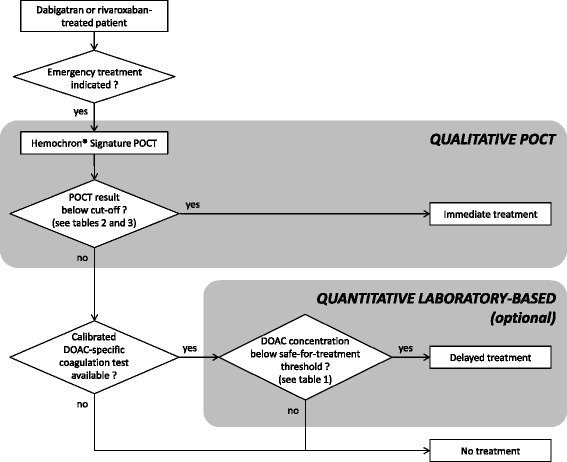
Proposed algorithm for emergency coagulation assessment in rivaroxaban- and dabigatran-treated patients. *DOAC* direct oral anticoagulant, *POCT* point-of-care test

**Fig. 4:**
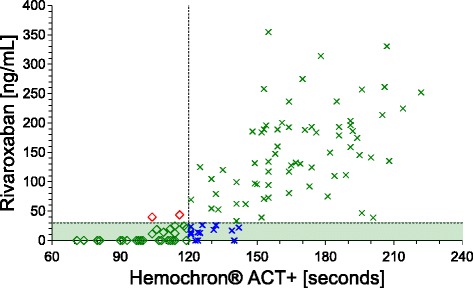
Scatter plot of rivaroxaban concentrations (*green area*: <30 ng/mL) and activated clotting time (*ACT+*) POCT illustrating the diagnostic accuracy of POCT results. *Green diamonds* represent samples below the safe-for-treatment threshold that are correctly identified as eligible for immediate treatment (true positive). *Blue crosses* represent samples that are not detected although containing concentrations below the safe-for-treatment threshold (false negative). These can potentially still receive delayed treatment if slower laboratory-based DOAC-specific tests are available. *Green crosses* represent samples above the POCT cut-off correctly identified as not eligible for treatment (true negative). *Red diamonds* represent the few samples incorrectly identified as eligible for treatment despite concentrations above the safe-for-treatment threshold (false positive)

According to the results of this study, Hemochron® Signature can be used to qualitatively rule out relevant concentrations of rivaroxaban and dabigatran during DOAC therapy, and accurately identify patients who are safe for surgery or thrombolysis. To ensure patient safety, we chose to establish cut-off values that provide >95% specificity. Although this limits the sensitivity of our results (i.e. not all eligible patients are identified), our approach allows the rapid identification of a relevant fraction of patients that can be treated immediately without the need to await the results of much slower laboratory-based tests (Figs. 3 and 4). For example, out of the 118 rivaroxaban samples included in this study, a Hemochron® Signature ACT+ test result ≤1.2 identified 34 samples as eligible for treatment based on a rivaroxaban threshold <30 ng/ml (Fig. 4). Out of these 34 samples, 31 were identified correctly (true positives, PPV 91%), demonstrating the high diagnostic accuracy. However, the limited sensitivity of our approach resulted in 15 samples that were not identified as eligible despite containing <30 ng/ml rivaroxaban (false negatives, NPV 82%).

Although both rivaroxaban and dabigatran can be detected with PT/INR or ACT+ POCT, our likelihood ratio calculations support the use of ACT+ test cards for rivaroxaban and PT/INR test cards for dabigatran.

Importantly, since there is no single POCT cut-off value that can safely rule out all DOAC, knowledge of the patient’s medication history is necessary. Furthermore, the blood concentration of all DOAC increase rapidly after intake. Therefore, coagulation results obtained within the first 4 h after intake are not reliable.

### Strengths and limitations

Our study adds considerable insight to previous reports. We are the first to evaluate the diagnostic accuracy of Hemochron® Signature to qualitatively rule out relevant DOAC concentrations of apixaban, rivaroxaban, and dabigatran. To the best of our knowledge, apixaban has never been tested with this device before. In a departure from previous studies, we aimed at establishing DOAC-specific cut-offs to provide clinicians with a practical tool for decision making during emergency treatment. All samples were acquired from real-life patients, avoiding the use of spiked plasma. Primarily collecting samples during treatment initiation rather than a steady state provided a high number of samples containing very low DOAC concentrations. Compared to previous studies [5–7], this allowed superior assessment of how such low concentrations influence coagulation assays. Due to low dabigatran concentrations in samples collected during treatment initiation, we decided to include samples from eight patients on maintenance therapy. Although this adds heterogeneity to our patient population, we did not find differences in correlation to POCT results between the two groups. Six sequential samples were acquired from each patient. Hence, a bias due to repeated measurements in individual patients cannot be excluded but this approach allowed us to cover a wide spectrum of DOAC concentrations. Due to the use of venous blood in this study, our results cannot be extended to test results obtained from capillary samples. However, as venous access is routinely obtained in all emergency patients we do not expect this to limit the clinical applicability of our results.

What constitutes a clinically relevant DOAC concentration is a matter for debate, and the concentration thresholds investigated in our study, albeit based on current literature, have not been confirmed by prospective clinical trials. Furthermore, generalizability of our results is limited by the single-centre nature of our trial. For these reasons, validation of our data is warranted, ideally including clinical outcome-oriented end-points.

## Conclusions

Our results suggest that Hemochron® Signature POCT in combination with PT/INR or ACT+ test cards can be used to qualitatively rule out relevant concentrations of rivaroxaban and dabigatran, but not of apixaban.

Thus, Hemochron® Signature POCT can be a fast and reliable alternative for guiding emergency treatment during rivaroxaban and dabigatran therapy. It allows the rapid identification of a relevant fraction of patients that can be treated immediately without the need to await the results of much slower laboratory-based coagulation tests. The high clinical relevance of this question warrants a large-scale trial to investigate the clinical safety of this approach.

## Additional file

Additional file 1: Table S1. Patient characteristics in the study groups. **Table S2.** Baseline laboratory results of patients in the study groups. **Table S3.** Diagnostic accuracy of Hemochron® Signature aPTT and ACT-LR POCT cards for dabigatran. (DOC 87 kb)

## Abbreviations

ACT: Activated clotting time
ACT+: Activated clotting time plus
ACT-LR: Activated clotting time-low range
aPTT: Activated partial thromboplastin time
DOAC: Direct oral anticoagulants
dTT: Diluted thrombin time
IQR: Interquartile range
LR: Likelihood ratio
NPV: Negative predictive value
POCT: Point-of-care testing
PPV: Positive predictive value
PT/INR: Prothrombin time/international normalized ratio
UPLC-MS: Ultra-performance liquid chromatography-tandem mass spectrometry
VKA: Vitamin K antagonists
