# Supplementary material for: Point-of-care testing for emergency assessment of coagulation in patients treated with direct oral anticoagulants
Source: Crit Care. 2017 Feb 15;21:32. doi: 10.1186/s13054-017-1619-z (PMC5309971; doi:10.1186/s13054-017-1619-z)
Supplement: Additional file 1: Table S1. — Patient characteristics in the study groups. Table S2. Baseline laboratory results of patients in the study groups. Table S3. Diagnostic accuracy of Hemochron® Signature aPTT and ACT-LR POCT cards for dabigatran. (DOC 87 kb) [file 13054_2017_1619_MOESM1_ESM.doc]

**Additional File 1**

**Table S1:** Patient characteristics in the study groups

| *Patient characteristic* | *Rivaroxaban* | *Apixaban* | *Dabigatran* | *Dabigatran (Maintenance Therapy)* |
| --- | --- | --- | --- | --- |
| Dose^1^ | 15mg: 2 (10%)  20mg: 18 (90%) | 2.5mg: 6 (30%)  5mg: 14 (70%) | 110mg: 5 (25%)  150mg: 15 (75%) | 110mg: 1 (13%)  150mg: 7 (88%) |
| Sex, female^1^ | 7 (35%) | 10 (50%) | 8 (40%) | 4 (50%) |
| Age, years^2^ | 69 ±14 | 74 ±13 | 70 ±13 | 64 ±11 |
| Body weight, kg^2^ | 81 ±19 | 68 ±22 | 82 ±26 | 89 ±19 |
| Body mass index^2^ | 27 ±6 | 25 ±2 | 28 ±7 | 30 ±5 |
| **Risk Factors** | | | |  |
| Arterial hypertension^1^ | 11 (55%) | 17 (85%) | 14 (70%) | 4 (50%) |
| Diabetes mellitus^1^ | 3 (15%) | 7 (35%) | 4 (20%) | 1 (13%) |
| Hyperlipidaemia^1^ | 5 (25%) | 13 (65%) | 14 (70%) | 3 (38%) |
| Smoking^1^ | 3 (15%) | 2 (10%) | 4 (20%) | 0 (0%) |
| **Concomitant antiplatelet agents (last dose <7 days)** | | | |  |
| Acetylsalicylic acid^1^ | 9 (45%) | 9 (45%) | 8 (40%) | 1 (13%) |
| Others^1^ | 1 (5%) | 1 (5%) | 0 (0%) | 0 (0%) |
| **Indication for oral anticoagulation** | | | |  |
| Atrial fibrillation^1^ | 12 (60%) | 5 (25%) | 18 (90%) | 7 (88%) |
| PFO^1^ | 8 (40%) | 0 (0%) | 2 (10%) | 0 (0%) |
| ESUS^1^ | 0 (0%) | 15 (75%) | 0 (0%) | 1 (13%) |

^1^number (%), ^2^mean ±standard deviation; ESUS, Embolic Stroke of Undetermined Source; PFO, Patent foramen ovale.

**Table S2:** Baseline laboratory results of patients in the study groups

| *Laboratory test* | *Rivaroxaban* | *Apixaban* | *Dabigatran* | *Dabigatran (Maintenance Therapy)* | *Reference Range* |
| --- | --- | --- | --- | --- | --- |
| WBC, /μl | 7233 ±2099 | 6815 ±1693 | 8097 ±2932 | 10341 ±1887 | 3800-10300 |
| RBC, 10^6/μl | 4.4 ±0.6 | 4.2 ±0.6 | 4.5 ±0.4 | 4.0 ±0.5 | 4.2-6.2 |
| Haematocrit, % | 40 ±5 | 38 ±5 | 40 ±4 | 36 ±4 | 42-52 |
| Haemoglobin, g/dL | 13.6 ±1.8 | 12.7 ±2.0 | 13.8 ±1.5 | 12.2 ±1.8 | 14.0-18.0 |
| Platelets, 10^3/μl | 231 ±49 | 250 ±75 | 227 ±79 | 202 ±51 | 150-450 |
| Quick, % | 99 ±12 | 96 ±14 | 95 ±12 | 73 ±13 | 70-120 |
| INR | 1.0 ±0.1 | 1.1 ±0.1 | 1.0 ±0.1 | 1.2 ±0.2 |  |
| aPTT, seconds | 25 ±3 | 28 ±5 | 27 ±4 | 43 ±11 | <40 |
| anti-Xa, IE-aXa/mL | <0.1 ±0 | <0.1 ±0 | <0.1 ±0 | <0.1 ±0 |  |
| Fibrinogen, mg/dL | 338 ±90 | 318 ±62 | 338 ±62 | 311 ±42 | 170-410 |
| D-dimer, μg/mL | 0.91 ±1.58 | 0.61 ±0.67 | 0.77 ±1.04 | 0.31 ±0.32 | <0.24 |
| Creatinine, mg/dL | 0.9 ±0.3 | 1.0 ±0.5 | 0.9 ±0.3 | 1.0 ±0.2 | 0.6-1.1 |
| GFR, mL/min/kg | 81 ±20 | 82 ±33 | 77 ±18 | 74 ±25 | >60 |
| Urea, mg/dL | 36 ±11 | 36 ±18 | 37 ±11 | 34 ±10 | 12-46 |
| Protein total, g/dL | 6.8 ±0.6 | 6.6 ±0.7 | 7.0 ±0.6 | 6.5 ±1.1 | 6.5-8.5 |
| Albumin, g/dL | 4.1 ±0.3 | 3.8 ±0.5 | 4.1 ±0.4 | 3.2 ±0.6 | 3.4-4.8 |
| CRP, mg/dL | 2.9 ±4.2 | 2.2 ±3.5 | 1.5 ±1.4 | 1.5 ±0.8 | <0.5 |
| Procalcitonin, ng/mL | 0.09 ±0.04 | 0.09 ±0.05 | 0.09 ±0.04 | 0.08 ±0.03 | ≤0.10 |
| AST, U/L | 35 ±12 | 37 ±20 | 37 ±19 | 45 ±22 | ≤50 |
| ALT, U/L | 35 ±19 | 36 ±35 | 34 ±22 | 32 ±21 | ≤50 |
| GGT, U/L | 48 ±37 | 42 ±44 | 68 ±76 | 56 ±29 | ≤60 |
| CHE, kU/L | 7.8 ±1.5 | 7.1 ±2.2 | 8.5 ±2.1 | 7.1 ±1.7 | 4.9-12.0 |

All results displayed in mean ±standard deviation; WBC, white blood count; RBC, red blood count; INR, international normalized ratio; aPTT, activated partial thromboplastin time; GFR, glomerular filtration rate; CRP, C-reactive protein; AST, aspartate transaminase; ALT, alanine transaminase; GGT, gamma-glutamyl transferase; CHE, cholinesterase.

**Table S3:** Diagnostic accuracy of Hemochron® Signature aPTT and ACT-LR POCT cards for dabigatran

| *Coagulation test result* | *Threshold (ng/mL)* | *Specificity (%)* | *Sensitivity (%)* | *LR* | *PPV (%)* | *NPV (%)* |
| --- | --- | --- | --- | --- | --- | --- |
| POCT  ACT-LR ≤95 seconds | <30 | 97 (89-100) | 18 (11-28) | 6.4 | 89 | 49 |
| POCT  ACT-LR ≤100 seconds | <50 | 96 (85-99) | 18 (12-27) | 4.5 | 91 | 35 |
| POCT  aPTT ≤60 seconds | <30 | 99 (91-100) | 34 (24-44) | 23.3 | 97 | 53 |
| POCT  aPTT ≤70 seconds | <50 | 96 (85-99) | 50 (40-59) | 11,9 | 97 | 45 |

Samples=168. Sensitivity and specificity are provided with 95% confidence intervals. ACT-LR indicates activated clotting time-low range; aPTT, activated thromboplastin time; LR, likelihood ratio; NPV, negative predictive value; POCT, point of care test; PPV, positive predictive value; PT, prothrombin time and TT, thrombin time.
